# Can We Put Aside Microelectrode Recordings in Deep Brain Stimulation Surgery?

**DOI:** 10.3390/brainsci10090571

**Published:** 2020-08-20

**Authors:** Jesús Pastor, Lorena Vega-Zelaya

**Affiliations:** Clinical Neurophysiology and Instituto de Investigación Biomédica, Hospital Universitario de La Princesa, C/Diego de León 62, 28006 Madrid, Spain; lorenacarolina.vega@salud.madrid.org

**Keywords:** extracellular recordings, imaging-guided surgery, intraoperative neurophysiology

## Abstract

Microelectrode recording (MER) in deep brain stimulation (DBS) surgery has long been a recognized and efficient method for defining a target. However, in recent decades, imaging techniques, including DBS surgery, have experienced significant growth. There is convincing evidence that imaging-guided surgery can be helpful for targeting anatomically well-defined nuclei (e.g., subthalamic nucleus (STN) or internal globus pallidus (GPi)), and reductions in secondary effects have also been claimed. It has even been proposed that MER is not necessary to perform DBS, identifying in this way asleep surgery and imaging-guided DBS. However, there are several reasons why this is not the case. Neurophysiological techniques can efficiently and safely help to identify neural structures even in sleeping patients (e.g., different types of evoked potentials or motor stimulation). Deep nuclei are not homogeneous structures (even STN), so it is important to identify different places inside the putative target. Evidently, this is more relevant in the case of thalamic or hypothalamic surgery. Moreover, it is important to remember that the clinical and scientific knowledge acquired during DBS surgery can be important to gain further insight into pathologies and develop more effective treatments. Finally, the cost/efficiency of medical technology should be considered.

Deep brain stimulation (DBS) is an effective and proven surgical treatment for several movement disorders (e.g., Parkinson disease (PD), essential tremor, or dystonia) and appears to be promising for other pathologies, such as epilepsy, pain, major depression, or Alzheimer’s disease [1]. The process for all these diseases is to implant electrodes at different targets to modify the pathological behavior of neural circuits by means of electrical stimulation. From the very beginning, microelectrode recording (MER) was a reliable technique to identify neural structures. This method contributed in an invaluable way to improve surgical outcomes and helped to offer theories about underlying pathophysiology [2,3,4], although there is still debate about its necessity [5,6,7].

In recent decades, imaging techniques have experienced an impressive expansion, colonizing even more hospital sites than was initially expected, such as operating rooms. As a natural consequence, computerized tomography scans (CT) and magnetic resonance imaging (MRI) not only have been used in oncological neurosurgery, but in functional brain surgery too, where the precise identification of deep targets is mandatory. The spreading of these methods has sparked a debate about the necessity of performing DBS on awake (under local anesthesia and able of conscious collaboration) or asleep (under general anesthesia) patients, even defining the imaging-based approach as a change in paradigm [7]. Sometimes, awake surgery has been identified as being neurophysiologically guided surgery, and asleep surgery as being imaging-guided.

It has been argued that imaging-guided surgery increased the targeting accuracy in subthalamic nucleus (STN) and internal globus pallidus (GPi) surgeries, improving the clinical outcomes in patients with PD; meanwhile, there were fewer complications than in awake patients operated under MER guidance [7]. On the contrary, there is also evidence that multisite MER increases outcomes in STN surgery for PD [4]. However, it has been reported that the use of MER is related to an increased risk of intracranial hemorrhage and cognitive decline [8]. Nevertheless, general anesthesia is not free of complications such as venous thromboembolism or pneumonia [9], and these risks should also be carefully evaluated.

In addition to concerns about the targeting accuracy and medical outcomes, the mental and clinical states of patients have been invoked to justify asleep surgery instead of awake [7]. Consideration of patient preferences and well-being should always be at the core of medical issues during surgery, and the reduction of stress and discomfort should prevail, favoring asleep instead of awake surgery [10]. Therefore, the true debate should not be asleep versus awake surgery, but whether we may dispense with MER and neurophysiological techniques during DBS surgery. Before an answer is stated, we must consider several facts.

Firstly, it must be considered that neurophysiological techniques, among which is MER, are able to identify safely and efficiently neural structures and functions even in sleeping patients. For example, electrical stimulation can estimate the distance of the inner capsule during STN or GPi surgery and visual evoked potentials can be used to identify optic pathways or somatosensory evoked potentials for medial lemniscus. Although it is well-known that general anesthesia modifies the pattern of discharge of neurons, it has been shown that, under some circumstances (i.e., using dexmedetomidine), MER can be safely used in sleeping patients [11,12].

Second, even if STN or GPi can be confidently identified by imaging, it is well known that basal ganglia circuits include different parallel systems, and single-cell recordings in animals have been shown to preserve functional specificity at the level of individual neurons throughout circuits [13], so the objective should not merely be to target STN or GPi, but to stimulate specific locations inside the nuclei. Moreover, some neural structures, such as the thalamus or the hypothalamus, are much more complex, lacking anatomical landmarks that discriminate between subnuclei. Although it has been proposed that diffusion tensor imaging can be useful in identifying white pathways [14], not all the relevant targets have these long pathways (e.g., the posterior hypothalamus). However, some thalamic [15,16] or hypothalamic [17] subnuclei show bioelectrical-specific features that allow a confident identification, even under general anesthesia. An unequivocal way to identify the thalamic ventrocaudal subnucleus is by the MER of somatosensory evoked potentials [18]. The positive identification of this structure helps to identify surrounding nuclei.

Third, there is a scientific consideration that we must keep in mind concerning MER. The anomalous functioning of the bioelectrical properties (i.e., the pattern of activity, timing, frequency, or synchronicity) of albeit morphologically normal neural networks is the most accepted theory regarding pathophysiology in most of these illnesses [19,20,21]. Therefore, the amount of scientific information we can obtain from the neurophysiological recordings from patients is invaluable. It is said that humans cannot be under any circumstance considered as experimental subjects. However, until MER is demonstrated (not suspected) as obsolete or deleterious for patients, we can increase our knowledge about several pathologies that can help better treat patients. Nowadays, we have a very promising technique for acquiring pathological information through DBS systems with recording features. This is an exciting opportunity for neuroscientists and clinicians; however, the electrode’s surface is adequate to record local field potentials, but not extracellular spikes. This information is probably enough to program electrical stimulation, but is insufficient for understanding pathology.

A final fourth issue that should be considered before changing the paradigm is the cost of equipment. Even in first-world countries, not all hospitals can afford to install an MRI in operating rooms. Medical treatments are more effective nowadays than at any other moment in history, but they are also much more expensive. Therefore, public health policies (at least in those countries where public universal access to health is sought) must carefully evaluate the necessity of costly investments, especially when effective and cheaper alternatives exist.

To summarize, we are far from demonstrating that MER is not needed for DBS surgery. While ethical concerns must be carefully considered, clinical trials could be accomplished to address this issue. This approach is mainly directed to evaluate clinical outcomes, risks and secondary effects. However, other aspects (such as clinical and scientific knowledge or economic cost) should be considered. Finally, the specific technique offered (asleep vs. awake or imaging-guided vs. neurophysiologically guided) will depend on several factors, among which are patient status and preferences, the training of clinical teams, and hospital facilities.

Therefore, the answer to the question raised in the title, in our opinion, should be a negative.
